# Prognostic Evaluation of Piezo2 Channels in Mammary Gland Carcinoma

**DOI:** 10.3390/cancers16132413

**Published:** 2024-06-29

**Authors:** Raquel Martín-Sanz, Aline Rodrigues-Françoso, Yolanda García-Mesa, Francisco Javier García-Alonso, María Asunción Gómez-Muñoz, Sandra Malmierca-González, Rocío Salazar-Blázquez, Olivia García-Suárez, Jorge Feito

**Affiliations:** 1 Instituto de Investigación Biomédica de Salamanca (IBSAL), 37007 Salamanca, Spain; rmartinsan@saludcastillayleon.es (R.M.-S.); sandramalmiercagonzalez@gmail.com (S.M.-G.); 2 Servicio de Oftalmología, Complejo Asistencial de Zamora, 49022 Zamora, Spain; 3 Servicio de Oncología, Complejo Asistencial Universitario de Salamanca, 37007 Salamanca, Spain; arodrigues@saludcastillayleon.es; 4 Grupo SINPOS, Department of Cell Biology and Morphology, University of Oviedo, 33003 Oviedo, Spain; garciamyolanda@uniovi.es (Y.G.-M.); garciaolivia@uniovi.es (O.G.-S.); 5 Instituto de Investigación Sanitaria del Principado de Asturias (ISPA), 33011 Oviedo, Spain; 6 Servicio de Digestivo, Hospital Universitario Rio Hortega, 47012 Valladolid, Spain; fgarciaalo@saludcastillayleon.es; 7 Servicio de Anatomía Patológica, Complejo Asistencial Universitario de Salamanca, 37007 Salamanca, Spain; asunciongomez@saludcastillayleon.es (M.A.G.-M.); rsalazarb@saludcastillayleon.es (R.S.-B.)

**Keywords:** Piezo2, piezo channels, mechano-signaling, breast cancer, immunohistochemistry

## Abstract

**Simple Summary:**

The expression of the mechanosensory Piezo2 channel has already been described in different malignant tumors. There is discordance in the literature regarding breast carcinoma, with its expression described either as decreased or increased in neoplasms with respect to benign tissue. A retrospective cohort of 125 patients whose breasts were resected for carcinoma was chosen to determine the relationship between Piezo2 and different clinical and histological variables. A significant association was found with the Ki67 proliferation index, with a tendency for most proliferative tumors to be positive for Piezo2.

**Abstract:**

In the last decade, a group of Ca^2+^ channels called Piezo were discovered, demonstrating a decisive role in the cellular response to mechanical stimuli and being essential in the biological behavior of cells regarding the extracellular compartment. Several investigations have suggested a potential role in carcinogenesis, with a tumor suppressor role in some cases but increased expression in several high-grade neoplasms. Regarding Piezo2 expression in mammary gland neoplasms, a protective role for Piezo2 was initially suggested, but a subsequent study demonstrated a relationship between Piezo2 expression and the highly aggressive triple-negative phenotype of breast carcinoma. A cohort of 125 patients with clinical follow-up was chosen to study Piezo2 expression and clarify its clinical implications using the same immunohistochemical evaluation performed for other breast carcinoma parameters. Fisher’s exact test was chosen to identify potential relationships between the different variables. A significant association was found with the Ki67 proliferation index, but not with mitoses. The tendency of most proliferative tumors was to have an increased score for Piezo2. A similar association was found between Piezo2 expression and perineural invasion.

## 1. Introduction

### 1.1. Piezo Channels

Piezo channels represent a new class of mechanosensitive channels, responding to mechanical stimuli by permitting Ca^2+^ ions to pass through the cell membrane to the cell´s cytoplasm, influencing the cell’s biology [1,2]. These channels were initially described in the endoplasmic reticulum membrane, also with a Ca^2+^ gradient due to lower concentrations in the cytoplasm [3], but posterior studies focused on their plasma cell membrane localization, conferring them mechanosensory ability [1,2]. Their opening leads to an increase in the cytoplasmic concentration of Ca^2+^ and, thus, to a transformation of tactile stimuli into action potentials [4]. The Piezo family consists of two ion channels with marked homology named Piezo1 and Piezo2 [5]. Mechanical forces stimulate these channels by the induction of a physical modification in the extracellular matrix that can pull or push the plasma membrane of the cell [6], tethered on the other side to the cell actin cytoskeleton [7,8]. Furthermore, integrin activation on the cell surface and adhesion to other cells and the extracellular matrix depend on Piezo channels [3].

Although Piezo2 is specifically related to mechanoreception in the Peripheral Nervous System [9,10], Piezo1 demonstrates a more ubiquitous distribution and a polymodal behavior. Piezo 1 channels are essential for vascular and erythrocyte function, osteoclastogenesis, and urinary excretion. They were also described as participating in biological processes associated with mechanical stimulation like vascular shear, urinary flow regulation, bladder distension, or volume regulation. Their function is generally related to elongation, cellular growth, migration, and proliferation [2,5]. Mutations in PIEZO2 can lead to neuromuscular syndromes, including distal arthrogryposis type 5, Gordon syndrome, and Marden–Walker syndrome [5].

### 1.2. Piezo and Cancer

Piezo mechanoreceptors were found in several tissues and neoplasms, but their function is not fully understood [2]. Mechanical stimuli may influence cancer biology, affecting both neoplastic cells and their environment, by altering cell migration, proliferation, matrix remodeling, and metastatic behavior [10]. In addition, Piezo channels have specific agonists (Yoda I) and antagonists (GsMTx4) that allow a precise study of this promising target [11].

Both Piezo1 and Piezo2 have diminished expression in lung neoplasms with respect to benign lung tissue, and a correlation was found between elevated Piezo mRNA expression and improved survival of non-small cell lung carcinoma [12]. An opposite behavior was found in urinary bladder and colonic human and murine neoplasms [13,14]. In gliomas, Piezo1 expression is increased in poorer prognostic neoplasms [15,16]. Moreover, increased Piezo1 expression was observed in metastatic colon carcinoma [17], prostate carcinoma [18], and oral squamous carcinoma [19].

One of the first known functions of Piezo channels was cellular adhesion through integrin activation [3], and this may explain why carcinomas, which are less cohesive and stationary than benign tissue, have, on some occasions, diminished Piezo expression and why higher-grade carcinomas (generally even less cohesive and indifferent to pressure made by surrounding cells and tissue) have much less expression [12]. The increased expression described in aggressive vesical neoplasms [13], high-grade gliomas [15,16], and others [17,18,19] may be more complex to explain; in this regard, it has been suggested that Piezo2 activation accelerates the cell cycle through activation of Akt/mTOR, enhancing the growth of the neoplasm [18].

In any case, both Piezo1 and Piezo2 have a potential role both as an oncoprotein and a tumor suppressor protein [11]. When dealing with a tactile-related protein such as this, it is unknown if physical perceptions of a tumor can change its fate. In this line, recent research shows that Piezo1 agonist Yoda1 is able to reduce macropinocytosis to impair cell nutrition [20].

### 1.3. Piezo and Mammary Gland Carcinoma

The presence of the Piezo2 channel has already been noted in epithelial cells of normal breasts, and current evidence suggests that Piezo2 is not limited to nervous tissue and probably has a wider function than Piezo1 [21]. In this regard, it has demonstrated expression at least in vascular structures and the different epithelial glandular components; a role of Piezo2 channels has been proposed in the milk-flow-induced response to duct shear and dilation through the gland, similarly to Piezo1 [21].

In breast carcinoma, Piezo1 and Piezo2 were studied in various different cell lines, with positive expression in all of them [22,23,24]. However, opposing results regarding Piezo1 and Piezo2 roles in breast cancer exist in the literature [22,23,24,25]. The first study, employing cell lines from normal breast and breast cancer, found increased Piezo1 expression in tumor cells compared with normal cells [23]. The second study indicated that Piezo2 expression is reduced in malignant cells, showing lesser expression as the tumor is more undifferentiated [22]. Moreover, the most recent study, focused on triple-negative breast cancer, found similar results to the Piezo1 study, as elevated *PIEZO2* mRNA expression was correlated with worse prognosis and lung metastases, finding no significant relation in hormone-positive carcinomas [25]. With the last interpretation [25], Piezo2 might be a biomarker of worse prognosis, but with the previous interpretation, it would be a biomarker of better prognosis [22]. So, discord exists in the bibliography regarding the prognostic relevance given to Piezo2 channels.

Currently, the classification of breast carcinoma includes the Nottingham score, which encompasses three histological features (pleomorphism, tubule formation, and mitoses) and the St. Gallen molecular phenotype classification [26,27,28]. The latter requires immunohistochemistry to classify carcinomas in luminal A, luminal B Her2+, luminal B Her2−, Her2+ non-luminal, and basal-like phenotypes.

The required immunohistochemistry includes hormonal receptors, Her2, and Ki67, for this purpose [27]. In breast, Ki67 is determinant in differentiating Luminal A from Luminal B subtypes, with a cut-off value of 20%. Ki67 is a nuclear protein characteristically expressed in proliferating vertebrate cells. This marker reacts with cells that are not in the G0 phase of the cell cycle and is commonly used in clinical cancer histopathology to assess the proliferation index [29,30,31,32]. For example, the Ki67 proliferation index is a determinant in GIST [33] and neuroendocrine tumors [34,35] and a valuable prognostic factor in other tumors like gliomas [36], lymphoma [37], sarcomas [38], melanoma [39], or carcinomas [40,41,42]. Additional prognostic features have been classically proposed regarding the epithelial-mesenchymal transition in different cancers [43]. In breast cancer, both perineural invasion [44] and tumor budding [45] have been noted as unfavorable.

To clarify the apparently opposing effects of Piezo2 channels and check their clinical relevance in breast cancer, we retrospectively chose a consecutive cohort of breast cancer patients undergoing surgery with a clinical follow-up of 5 years. Piezo2 immunohistochemistry was performed in the cohort similar to the commonly performed progesterone and estrogen receptors (PR and ER, respectively). The study included the relationship between Piezo2 expression and several clinical and histological features, including the Nottingham score and the St. Gallen molecular phenotype classification [26,27,28].

## 2. Materials and Methods

### 2.1. Patients

The medical records of 125 consecutive patients undergoing surgery for breast carcinoma treated between 2012 and 2013 in a single center were collected (Table 1). It mostly included patients with ductal adenocarcinoma, but other diagnoses were not excluded. A total of 114 patients had invasive carcinoma, and 11 patients had in situ carcinoma. Three (1 ductal in situ and 2 invasive ductal adenocarcinoma) samples were deemed invalid because of insufficient material in the remaining paraffin block. The margins were complete in all the tumors, with no evidence of neoplastic remnants. If a patient presented with two synchronous tumors, the one with the one with the highest grade was considered. A total of 63 patients had lymph node metastases at the time of diagnosis.

The histologic grade, according to the Nottingham classification, was available in a total of 109 invasive cases. According to this classification, a score of 1 to 3 was assigned to the different categories, including pleomorphism, tubule formation, and mitosis. These were added to obtain the grades (grade 1 with a score of 3–5; grade 2 with a score of 6–7; grade 3 with a score of 8–9). This information is quantified in Table 2.

Immunohistochemistry was available in most neoplasms (Table 3), and the St. Gallen subtypes were determined according to the immunohistochemical profile regarding hormone receptors, Her2, and Ki67 proliferation index (Table 4). Original Ki67 slides were retrieved; the immunohistochemical assay employed the Leica Bond automated platform (Leica Microsystems, Newcastle Upon-Tyne, UK) with SP6 clone anti-Ki67 antibodies (Master Diagnostica, Granada, Spain), diluted 1:100. Included subtypes were Luminal A (ER and/or PR+, Her2− and Ki67 < 30%), Luminal B Her2− (ER and/or PR+, Her2− and Ki67 ≥ 30%), Luminal B Her2+ (ER and/or PR+ and Her2+), Her2+ non luminal (ER/PR−, Her2+), and Basal-like (ER/PR−, Her2−).

The follow-up of the patients was at least 5 years after diagnosis. Possible clinical events were correlated with clinical progression of the neoplasm (relapse, metastases, etc.).

### 2.2. Immunohistochemical Assay

The paraffin blocks and slides where immunohistochemistry was performed at diagnosis were retrieved in all the cases. A tissue microarray was considered, but finally avoided in order to identify possible intra-tumoral heterogeneity of immunostaining. Moreover, benign breast tissue was present in 61 cases in addition to the neoplastic tissue.

Deparaffinized and rehydrated 5 µm sections were processed for detection of Piezo2 using the EnVision antibody complex detection kit (Dako^®^, Copenhagen, Denmark) following the supplier’s instructions. Briefly, endogenous peroxidase activity was inhibited (3% H_2_O_2_ for 15 min), then buffer was applied for 15 min (Leica Bond wash solution, Leica Biosystems^®^, Newcastle upon Tyne, UK), and non-specific binding was blocked (10% bovine serum albumin for 20 min). Sections were then incubated overnight at 4 °C with the primary antibody. The antibody against Piezo2 was polyclonal raised in rabbit (HPA040616, Sigma-Aldrich^®^, Madrid, Spain); it was used diluted to 1:200. The tuning of the antibody discarded non-specific binding employing bovine fetal serum (Sigma-Aldrich^®^, Madrid, Spain) instead of the primary and secondary antibodies. There are several articles in the bibliography endorsing the use of this antibody for Piezo2 [9,21,46,47,48,49]. Subsequently, the sections were incubated with anti-rabbit EnVision system-labeled polymer (Dako^®^, Copenhagen, Denmark) for 30 min. Finally, the slides were washed with buffer solution, and the immunoreaction was visualized with diaminobenzidine as a chromogen and washed. To ascertain structural details, the sections were counterstained with Mayer’s haematoxylin and finally dehydrated and mounted with Entellan (Merck^®^, Dramstadt, Germany). We also performed immunohistochemistry targeted against Piezo1 (PA5-72974, Invitrogen, Waltham, MA, USA), but we were not able to obtain clear images of expression in the breast specimens.

Quantification of the immunohistochemical expression of Piezo2 channels was performed according to Allred score, commonly used in evaluating breast hormone receptors [26,50]. This score assesses intensity of stain (0–3 points: negative, +, ++, or +++) and the proportion of positive neoplastic cells (0–5 points: negative, <1%, 1–10%, 11–33%, 34–66%, >66%). The two components are added for a total of 0–8 points. Although this scoring system can deliver biologically inconsistent results because of the addition of frequency and intensity of immunomarking in the same score, the wide clinical use regarding breast pathology made us choose it. Two different pathologists independently examined all the samples and reached a consensus afterwards. Images of the immunohistochemical results were taken with a Nikon Eclipse Ci microscope paired with a Nikon DS-Ri2 camera and employing Nikon NIS Elements F software, version 5.21.00 (Nikon^®^, Tokio, Japan).

### 2.3. Data Analysis

The statistical analysis was performed using the Stata package v. 13 (2013; StataCorp, College Station, TX, USA). Categorical variables are reported as percentages. Normally distributed continuous variables are summarized using means with standard deviations. Non-normally distributed continuous variables are reported as medians and interquartile ranges. The Pearson χ^2^ test and Fisher exact tests were used to assess differences between categorical values as warranted. Interrater agreement was assessed using Cohen’s kappa. The kappa result was interpreted as follows: 0–0.20 indicates no agreement, 0.21–0.39 indicates minimal agreement, 0.40–0.59 indicates a weak agreement, 0.60–0.79 indicates moderate agreement, 0.80–0.90 indicates strong agreement, and any value above 0.90 indicates an almost perfect agreement [51].

## 3. Results

### 3.1. Immunohistochemistry

**Figure 1:**
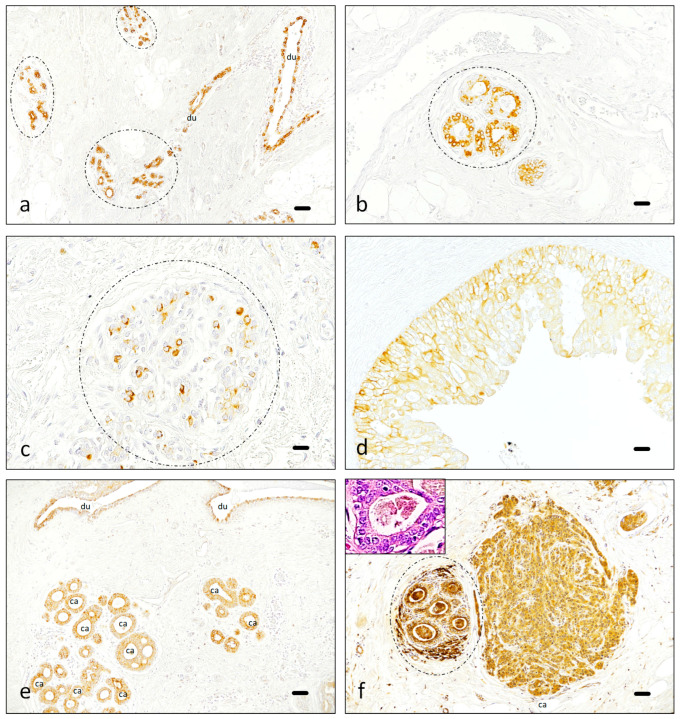
Piezo2 expression in non-neoplastic conditions. In most ducts (du) and acini (discontinuous circle), Piezo2 expression is positive in part of the epitelial cells (**a**–**c**). Benign hyperplastic regions tend to have a wider expression, staining all or nearly all the cells (**d**). In situ (**e**) and invasive carcinoma (**f**) are usually uniformly positive (ca in images (**e**,**f**)), contrasting with the scattered positive cells present in benign ducts (du in image (**e**)) and acini (discontinuous circle in image (**f**)), where nonspecific staining of mammary secretion is also observed (the insert in image (**f**) corresponds to a benign gland stained with hematoxylin-eosin illustrating secretion debris in the same patient). Piezo2 is sometimes intense in peri-lobular fibroblast-appearing cells of the connective tissue surrounding mammary glands in close proximity to the carcinoma (**f**). Scale bar 100 µm (**a**,**e**,**f**), 50 µm (**b**,**d**), 25 µm (**c**).

Available normal breast tissue usually showed intense, generally scattered, Piezo2 expression in glandular cells, which served as a positive control (Figure 1a–c). The immunostaining in glandular cells was variable, sometimes as rows of positive epithelial cells with few negative cells in the gland (Figure 1a,b) and mostly as single positive cells surrounded by abundant negative epithelial cells (Figure 1c). On the other hand, results in neoplastic tissue were commonly slightly less intense, and the expression was commonly homogeneous in either benign proliferative conditions (Figure 1d) or malignant neoplasms (Figure 1e,f). An additional finding was the presence of Piezo2 and several positive fibroblast-appearing cells in the connective tissue surrounding some benign glands (Figure 1f). Negative control is present in the mature fibrous areas surrounding benign glandular tissue (Figure 1).

**Figure 2:**
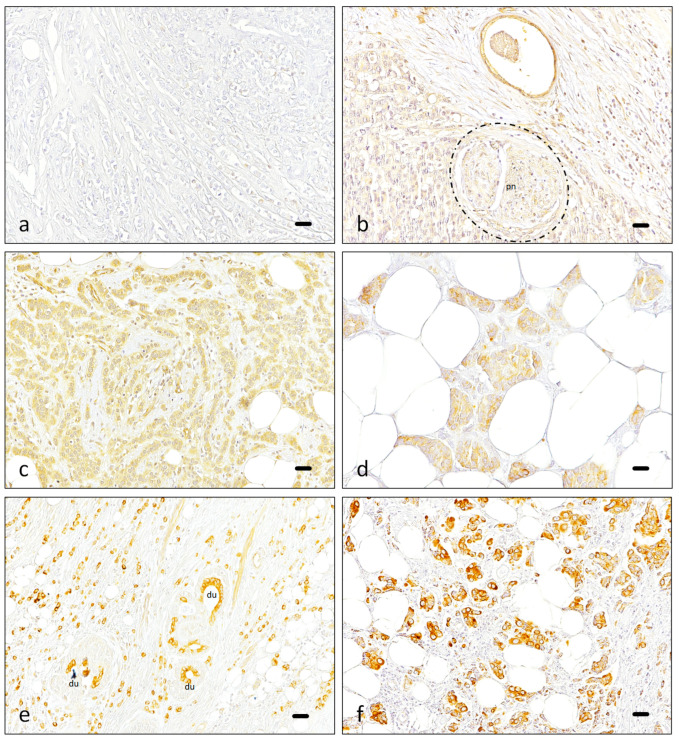
Piezo2 expression in neoplastic conditions. Images illustrating the different scores applied to the neoplastic cells: Negative (**a**), 1+ (**b**), 2+ (**c**,**d**) and 3+ (**e**,**f**). Sometimes, additional features were noted, like perineural invasion (pn in (**b**), nerve is highlighted with a discontinuous line) or remnants of benign mammary tissue inside the neoplasm (du in (**e**)). Scale bar 50 µm (**a**–**c**,**e**,**f**), 25 µm (**d**).

Neoplastic tissue was evaluated by two pathologists (Figure 2). A 90.5% agreement was observed between both in the first examination, with a Cohen´s kappa of 0.59 (95% CI 0.30–0.87) (Supplementary Materials). An assessment of controversial cases showed the most divergent opinions were related to the interpretation of background staining.

The majority of cases were positive for Piezo2, with variable pattern and intensity, with score 8 being the most frequent category, with intense and either complete or nearly complete expression in the neoplasm (Table 5).

**Figure 3:**
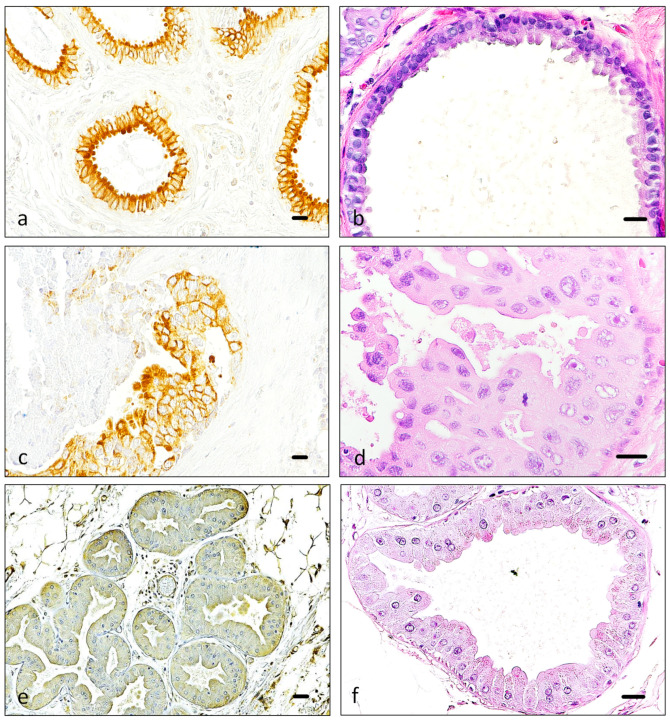
Piezo2 luminal expression pattern. Piezo2 immunostaining (**a**,**c**,**e**) and hematoxylin-eosin staining (**b**,**d**,**f**) illustrate the same region of three different patients with higher magnification ((**a**,**b**), (**c**,**d**) and (**e**,**f**)). When mammary secretion was prominent in benign tissue, in the form of apical apocrine-like “snouts”, Piezo2 expression was also intense in this region of the cell cytoplasm (**a**,**b**). Sometimes, when the malignant neoplastic tissue maintains good secretory differentiation and snouts are recognizable, Piezo2 expression is also highlighted in the luminal region of the cell (**c**,**d**). Areas of well-defined apocrine metaplasia are mostly negative with Piezo2 (**e**,**f**). Scale bar 50 µm (**a**–**f**).

In addition, a particular staining pattern was observed when the epithelial cells showed intense secretory activity (luminal or apical “snouts”, usual in apocrine-type secretion). This activity was sometimes accompanied by intense Piezo2 expression, both in benign (Figure 3a,b) and malignant tissue (Figure 3c). Genuine apocrine metaplasia is morphologically accompanied by an increase in size and intensely eosinophilic cytoplasms, in addition to the subtle apocrine snouts. When normal mammary gland tissue was noted as “apocrine” and snouts were less apparent, Piezo2 staining was commonly faint (Figure 3d).

### 3.2. Data Analysis

**Figure 4:**
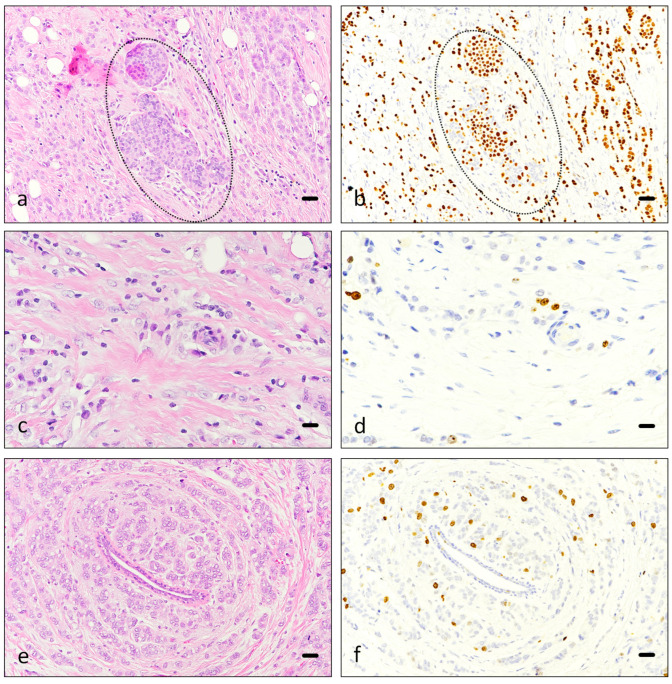
Ki67 immunostaining. Hematoxylin-eosin (**a**,**c**,**e**) and Ki67 immunostaining (**b**,**d**,**f**) of breast carcinoma, corresponding to consecutive slides ((**a**,**b**), (**c**,**d**) and (**e**,**f**)). Images (**a**,**b**) correspond to an infiltrating lobular carcinoma, including in situ carcinoma component (discontinuous line). Images (**c**,**d**) and (**e**,**f**) depict ductal carcinoma in two different patients. A benign duct is centrally located in images (**e**,**f**). Scale bar 50 µm (**a**–**f**).

Immunohistochemical results indicate that benign breast tissue demonstrated greater intensity of staining for Piezo2 than neoplastic tissue in the 61 cases where neoplastic and benign breast tissue were present in the same slide, with a median intensity of 2 for neoplastic breast tissue and a median of 3 for normal mammary glands (Figure 4) (Table 6). With non-parametric tests, the group of neoplastic tissue presented greater values (*p* = 0.004). Only three cases had apocrine metaplasia in normal mammary glands, all of them with an intensity of 1 for Piezo2.

Fisher´s exact test revealed no significant correlation between Piezo2 expression and survival, the tumor’s histology, stage, tubule formation, pleomorphism, mitoses, infiltration, or lymph node invasion. No relationship was observed between Piezo2 and ER, PR, or Her2. The relationship of Piezo2 with mitoses revealed a Fisher´s exact *p* = 0.06, but when categorizing Piezo2 under two categories (score < 2 and ≥2), the Fisher’s exact was 0.81.

**Figure 5:**
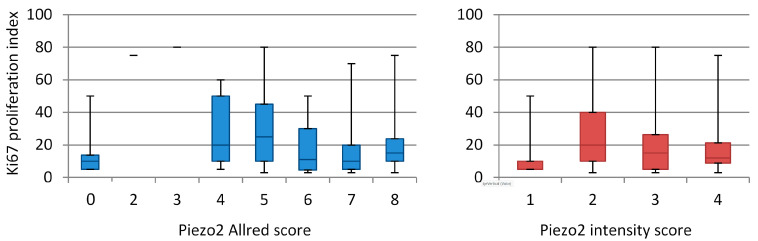
Piezo2 expression in relation to Ki67 proliferation index. Global Allred score for Piezo2 is depicted on the horizontal axis in the left diagram, where only one case was present in categories 2 and 3 of Piezo2 Allred score. Right diagram illustrates only the Piezo2 intensity score component, separated from the extent of immunostaining. The cohorts are divided into quartiles and related to the Ki67 proliferation index on the vertical axis.

In contrast, a clear relationship was identified between Piezo2 expression and Ki67, with *p*-values under 0.05, with higher Piezo2 expression in proliferative tumors. This finding was significant in categorizing Ki67 in two categories (≥20% and <20%) and in three categories (≥30%, >5%, <30%, and ≤5%). In the first case, the Fisher´s exact test *p* value was 0,01, and in the second one, it was 0,02. The relationship was confirmed when the expression of Piezo2 was divided into two categories (<2 and ≥2), also categorizing Ki67 in the previous two categories (*p* = 0.01). Results for Piezo2 and Ki67 in the studied cohort are summarized in Figure 5, and the precise Ki67 and Piezo2 categorized results are included as Supplementary Materials.

**Figure 6:**
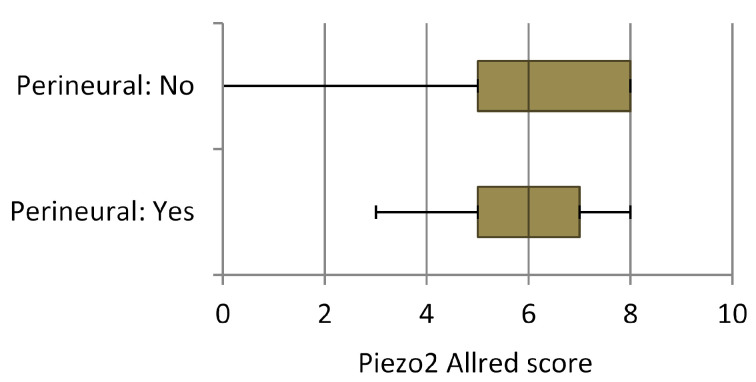
Piezo2 expression in relation to perineural infiltration. The global Allred score for Piezo2 is illustrated on the horizontal axis. The patients are divided into quartiles.

A similar relationship was found regarding perineural invasion, with a Fisher´s exact test’s *p* value of 0.01. The number of cases noted as positive for perineural infiltration was 15, and the number without it was 97 (Figure 6).

## 4. Discussion

In our experiment, a majority of breast cancer patients were positive for Piezo2, which is in accordance with the literature [22]. The apparent discordance between the studies regarding Piezo1 and Piezo2 correlation with aggressive phenotypes in breast neoplasms might be partly explained by the limited representativeness of the MCF-7 breast cancer cell line employed in the older study [23,52]. However, other studies employing different lines have produced similar results, indicating an association between *PIEZO1* and poor prognosis [53,54], probably through the induction of a compression-enhanced invasive phenotype and matrix degradation of cancer cells through Piezo1 channels [55,56]. Moreover, the MCF-7 cell line was also employed in the first study regarding Piezo2 expression, with opposite results to Piezo1 regarding the prognostic significance, suggesting a relationship between Piezo2 and the least aggressive neoplasms [22]. Although both Piezo channels have high homology, their functional responses are different [56], and for this reason, Piezo1 determines a poor prognosis in the MCF-7 line while Piezo2 determines the opposite results in the same cell line [22,23]. It is accepted that extracellular matrix stiffening may induce the opening of these channels, increasing the cytoplasmic Ca^2+^ concentration [57], Piezo1 being activated either by positive or negative pressure over the cellular membrane, and Piezo2 being activated only by positive pressure [58]. The biological consequences of the opening of both Piezo channels are probably similar and related to the oncogenic AKT/mTOR pathways [18]. Although the mTOR pathway seems to be related to Ca^2+^ mobilization, this relationship is not fully understood [59], and the association between Ca^2+^ and and AKT signaling is still unclear in breast cancer cells [60]. The presence of Piezo2-positive fibroblasts in the connective tissue surrounding some benign glands close to the carcinoma may be related to a neoplastic induction of a desmoplastic reaction and the delay of the healing process by non-tumor fibroblasts, as long as both Piezo channels are described to delay fibroblastic healing/scar processes [61].

Transcriptomics indicate that *PIEZO2* is particularly abundant in proprioceptors and sensory structures, extending to other organs including the lungs, urinary bladder, and gut [62]. The Human Protein Atlas indicates immunohistochemical expression in benign and malignant tissues, considering PIEZO2 as not prognostic in breast cancer. The Human Protein Atlas also has RNAseq data, with an average FPKM count of 2.1 for breast cancer and an average nTPM consensus of 1.2 for normal breast tissue. Although the counting method is not the same, it can be suggested that there is an increased expression of PIEZO2 in breast carcinoma [63,64]. This finding is in discordance with the findings in our series of 125 patients, in which increased Piezo2 was observed in benign breast tissue compared with neoplastic tissue. It is also in discordance with the previous work studying cell lines, which found increased expression in benign breast epithelial cells [22]. Discordance might be explained by the pattern of expression of Piezo2, with less-intense but homogenous immunostaining in neoplastic tissue and intense but with an interspersed pattern in benign tissue. Homogenization of tissue related to molecular quantification may contribute to the Human Protein Atlas results. This way, if benign tissue has Piezo2 expression, it would be of higher intensity, but, globally, malignant tissue would have more Piezo2 expression due to its clonal uniformity. The Human Protein Atlas refers to opposite results for PIEZO1, with an average FPKM count of 12.3 in breast cancer and an average nTPM count of 63.0 for benign breast tissue; Piezo1 was not detected in the Atlas employing antibody staining [63,64] and was not detected in our unpublished data.

Although we found stronger Piezo2 expression in benign breast tissue, we also detected a global tendency for more Piezo2 expression in the more proliferative neoplasms. This may be the key point to explain the apparent discordance mentioned in the introduction regarding the different studies employing cellular lines to study Piezo2 expression [22]. With this interpretation, Piezo2 would have diminished expression in carcinomas, but its expression would be enhanced in moderately or highly proliferative carcinomas, as measured by the Ki67 immunostaining, in line with the findings of Katsuta et al. [25]. However, a striking finding in the present study is that the highest proliferation was observed with a mild-to-moderate (2–5) Allred score for Piezo2 and a mild (1+) Piezo2 intensity score, as illustrated in Figure 5. This apparently contradictory clinical/pathological finding remains to be further explored because, although Piezo2 overexpression was already noted in aggressive carcinomas [13,14,15,16,17,18,19,25], this is the first study including a cohort of more than 100 patients with complete morphological and clinical evaluation.

We found a relevant relationship between Piezo2 expression and the Ki67 proliferation index, but the relationship with mitosis was only nearly relevant (*p* = 0.06) when dividing the sample into eight categories. It became irrelevant, reducing Piezo2 expression into two categories. By probably increasing the sample size and considering different cut-off values, a significant relationship might be demonstrated. In any case, mitosis counting is quite related to the Ki67 proliferation index [39]. In breast carcinoma, inconsistencies have been described between elevated Ki67 index and high mitosis accounts [30,32,65], but Ki67 remains a strong prognostic factor and is closely linked with mitotic count [32,66,67]. It has been described as the most powerful IHC prognostic indicator of early breast cancer in univariate analyses, probably due to its correlation with tumor grade [68]. Furthermore, the mitosis and Ki67 relationship in breasts varies dynamically, and the mentioned inconsistencies were related to certain cancer subtypes [32,69]. An explanation of this behavior may be the parallel relationship existing between the immune response against cancer cells and the actual proliferation of neoplastic cells, both requiring the existence of Ki67, which enables the proliferation of both immune and neoplastic cells [31]. The most undifferentiated tumors, generally with higher Ki67 expression and increased mutational burden in the neoplastic cells, may also have mutated immune control points, leading to a decreased immunological response against the cancer cells, thus escaping from the immunological response [30]. Piezo1 has also been described as affecting the immune response by enhancing myeloid-derived suppressor cells in cancer and infectious diseases, inhibiting immune responses, and, thus, promoting cancer proliferation [70].

Although mitosis is one of the three items included in the Nottingham histological grading system, Ki67 has a predictive role and has even been suggested as a criterion to select the therapy in some breast carcinomas [71]. In addition, Ki67 evaluation is critical, according to the St. Gallen Consensus, differentiating Luminal A and Luminal B molecular subtypes [30]. We have found a significant relationship between Piezo2 expression and the Ki67 proliferation index, considering Ki67 under two or three categories. The categorization of Ki67 into two categories was employed by many authors and is associated with the St. Gallen breast cancer categories and overall survival [72,73]. Although the cut-point selection ranges from 15% to 20%, in clinical practice, the Ki67 proliferation index is generally semiquantitatively measured and reported in 5% intervals, so, actually, breakpoints of >15% and ≥20% are actually similar and coherent with the St. Gallen Consensus. A recent study including more than 80.000 patients found that a Ki67 count of more than 20% confers a higher risk of distant metastasis, similarly to hormone receptor negative status or advanced stage [74]. The categorization of Ki67 under three categories is probably the best option for early breast cancer, as long as it renders more predictive value to the extreme categories <5% and >30% [30,66,71]. For this reason, two Ki67 categorizations were employed in the statistical analysis, both of which were significant. In any case, uncertainty remains surrounding the selection of relevant cut-off points for Ki67 [30,32]. The Nottingham histological grading system has good inter- and intra-observer agreement; it demonstrated strong association with patients´ survival and metastasis and is endorsed by the WHO and the College of American Pathologists [26,75]. However, for treatment purposes, Luminal A and Luminal B Her2+ are included in the Hormone Receptors Positive/Her2 Positive category [74]. The Nottingham score employs mitosis count in one of its three included items, but it has a low concordance rate among pathologists and lacks evidence of prognostic significance as a stand-alone parameter in breast cancer. For these reasons, Ki67 can better represent proliferative activity and attracts more attention as a measure of proliferation [32].

The main limitations in the present study are the sample size and, particularly, the tuning of the immunohistochemistry for Piezo2. Although our group has experience with this immunohistochemical technique, the background staining prevented a high level of concordance between observers. In any case, certain variability in the scoring by histopathologists appears when different individuals make interpretations over a slide [30]. Moreover, no specific threshold has been established for this technique, and the use of the Allred score was arbitrarily chosen only for its wide application. For this reason, this can only be considered a preliminary study. Other considerations to be made are related to the retrospective nature of the investigation, but we think that this approach was necessary to find some preliminary results that may guide a subsequent prospective study.

## 5. Conclusions

This is the first study assessing Piezo2 expression in breast carcinoma employing clinical data from a cohort of patients. Including the above-mentioned limitations, normal breast tissue showed enhanced Piezo2 expression when compared with neoplastic tissue. On the other hand, a significant positive relationship was demonstrated between Piezo2 expression, elevated Ki67 proliferation index, and perineural invasion. Moreover, there is a morphological shift from the single-cell expression of benign tissue to a more generalized expression in neoplasms.

## Figures and Tables

**Table 1:** Histology (**a**) and stage (**b**) of the cases. Ductal/Lobular category refers to a case with two adjacent tumors in the same breast quadrant (each one > 1 cm).

**a**		
**Histology**	**Number**	**Percent**
Ductal	105	84
Lobulillar	9	7.2
Mucinous	4	3.2
Turbomolecular	2	1.6
Dutal/lobulillar	1	0.8
Dutal + tubular	1	0.8
Medullary	1	0.8
Micropapillary	1	0.8
Solid papillary	1	0.8
**b**		
**Stage**	**Number**	**Percent**
pTis	8	6.4
pT1a	2	1.6
pT1b	18	14.4
pT1c	62	49.6
pT2	31	24.8
pT3	2	1.6
pT4	1	0.8

**Table 2:** Nottingham grade (**a**) and histologic features (**b**–**d**).

**a**	**Nottingham grade**	**Frequency**	**Percent**
	Grade 1	32	29.36
	Grade 2	43	39.45
	Grade 3	34	31.19
**b**	**Tubule formation**	**Frequency**	**Percent**
	Score 1	8	7.34
	Score 2	20	18.35
	Score 3	81	74.31
**c**	**Pleomorphism**	**Frequency**	**Percent**
	Score 1	13	11.93
	Score 2	68	62.39
	Score 3	28	25.69
**d**	**Mitoses**	**Frequency**	**Percent**
	Score 1	66	60.55
	Score 2	26	23.85
	Score 3	17	15.6

**Table 3:** Immunohistochemistry of breast carcinoma.

** Estrogen Receptor **	** Frequency **	** Percent **	** Progesterone Receptor **	** Frequency **	** Percent **
Positive	106	84.8%	Positive	85	68%
Negative	19	15.2%	Negative	45	32%
** Hormone Receptors **	** Frequency **	** Percent **	** Her2 **	** Frequency **	** Percent **
Positive	108	86.4%	Positive	36	29%
Negative	17	13.6%	Negative	88	71%

**Table 4:** St. Gallen classification.

St. Gallen Classification	Frequency	Percent
Luminal A	69	55.2%
Luminal B Her2−	15	12%
Luminal B Her2+	24	19.2%
Her2+ non luminal	11	8.8%
Basal-like	6	4.8%

**Table 5:** Piezo2 expression in infiltrating carcinoma.

Cells Expressing	<1% (1)	1–10% (2)	11–33% (3)	34–66% (4)	>66% (5)
Low expression (1)	1	2	9	14	17
Medium expression (2)	0	0	6	6	17
Intense expression (3)	0	0	0	3	36

Percentage of Piezo2-expressing tumor cells is depicted in the columns. The intensity of the expression is depicted in the rows. The score granted, according to the Allred score system, in specified between parentheses for all categories.

**Table 6:** Piezo2 intensity in normal breast and cancer.

Normal Gland			Cancer		
Value	Frequency	Percent	Value	Frequency	Percent
Score 0	1	1.6%	Score 0	5	8.2%
Score 1	16	26.2%	Score 1	22	36.1%
Score 2	12	19.7%	Score 2	18	29.5%
Score 3	32	52.5%	Score 3	16	26.2%

## Data Availability

Data is maintained within this article.

## Supplementary Materials

The following supporting information can be downloaded at: https://www.mdpi.com/article/10.3390/cancers16132413/s1, SM file: Ki67 and Piezo2 results in the cohort. Figure S1: Consecutive sections of breast carcinoma stained with H-E, Ki67, and Piezo2.
