# Supplementary material for: Prognostic Evaluation of Piezo2 Channels in Mammary Gland Carcinoma
Source: Cancers (Basel). 2024 Jun 29;16(13):2413. doi: 10.3390/cancers16132413 (PMC11240440; doi:10.3390/cancers16132413)
Supplement: Supplementary file 1 [file cancers-16-02413-s001.zip › Ki67 results v1.pdf]

Exact Ki67 measure values per category.

Ki67 in Piezo2 = 0: 5, 10, 20, 10, 5, 50, 5, 15, 10, 5, 10, 10 (12): mean 12,92%

Ki67 in Piezo2 = 2: 75 (1): mean 75%

Ki67 in Piezo2 = 3: 80 (1): mean 80%

Ki67 in Piezo2 = 4: 5, 50, 20, 10, 60, 15, 20 (7): mean 25,71%

Ki67 in Piezo2 = 5: 20, 8, 25, 70, 25, 40, 40, 50, 10, 60, 80, 20, 10, 50, 40, 15, 10, 25, 3, 10, 30 (21): mean 30,52%

Ki67 in Piezo2 = 6: 5, 30, 12, 3, 20, 30, 25, 50, 25, 3, 3, 25, 50, 10, 30, 10, 3, 5, 40, 10, 5, 3 (22): mean 18,05%

Ki67 in Piezo2 = 7: 10, 20, 10, 10, 30, 40, 3, 20, 5, 10, 70, 15, 3, 5, 5, 10, 10, 5, 15 (19): mean 15,58%

Ki67 in Piezo2 = 8: 5, 30, 5, 10, 20, 40, 15, 25, 15, 15, 12, 15, 25, 10, 12, 40, 20, 75, 5, 20, 25, 3, 3, 20, 5, 3, 10, 20, 30, 60, 3, 10, 10, 10, 15, 10 (36): mean 18,08%

119 cases

Ki67 in Piezo2 intensity = 0: 5, 10, 10, 5, 50, 10, 5, 10 (9-1): mean 13,13%

Ki67 in Piezo2 intensity = 1: 5, 20, 50, 8, 25, 70, 20, 40, 30, 12, 60, 75, 30, 20, 10, 10, 50, 25, 3, 3, 40, 60, 50, 15, 15, 10, 30, 80, 10, 3, 5, 25, 3, 40, 30, 40, 10, 10, 5, 30 (43-3): mean 26,93%

Ki67 in Piezo2 intensity = 2: 10, 20, 25, 10, 30, 40, 50, 5, 40, 20, 10, 3, 5, 70, 15, 20, 3, 5, 5, 10, 15, 10, 5, 10, 15 (30-4): mean 17,35%

Ki67 in Piezo2 intensity = 3: 5, 30, 10, 5, 10, 20, 40, 15, 25, 15, 15, 12, 3, 15, 25, 10, 40, 20, 75, 5, 20, 25, 3, 10, 3, 20, 5, 3, 10, 20, 30, 60, 3, 10, 10, 10 (38-3): mean 18,2%

In situ carcinoma:

Piezo2 intensity = 0: 10

Piezo2 intensity = 1: 25, 10, 20

Piezo2 intensity = 2: 25, 20, 80, 50, 3

Piezo2 intensity = 3: 12, 10
