# Supplementary material for: Prognostic Evaluation of Piezo2 Channels in Mammary Gland Carcinoma
Source: Cancers (Basel). 2024 Jun 29;16(13):2413. doi: 10.3390/cancers16132413 (PMC11240440; doi:10.3390/cancers16132413)
Supplement: Supplementary file 1 [file cancers-16-02413-s001.zip › supplemental figure.pdf]

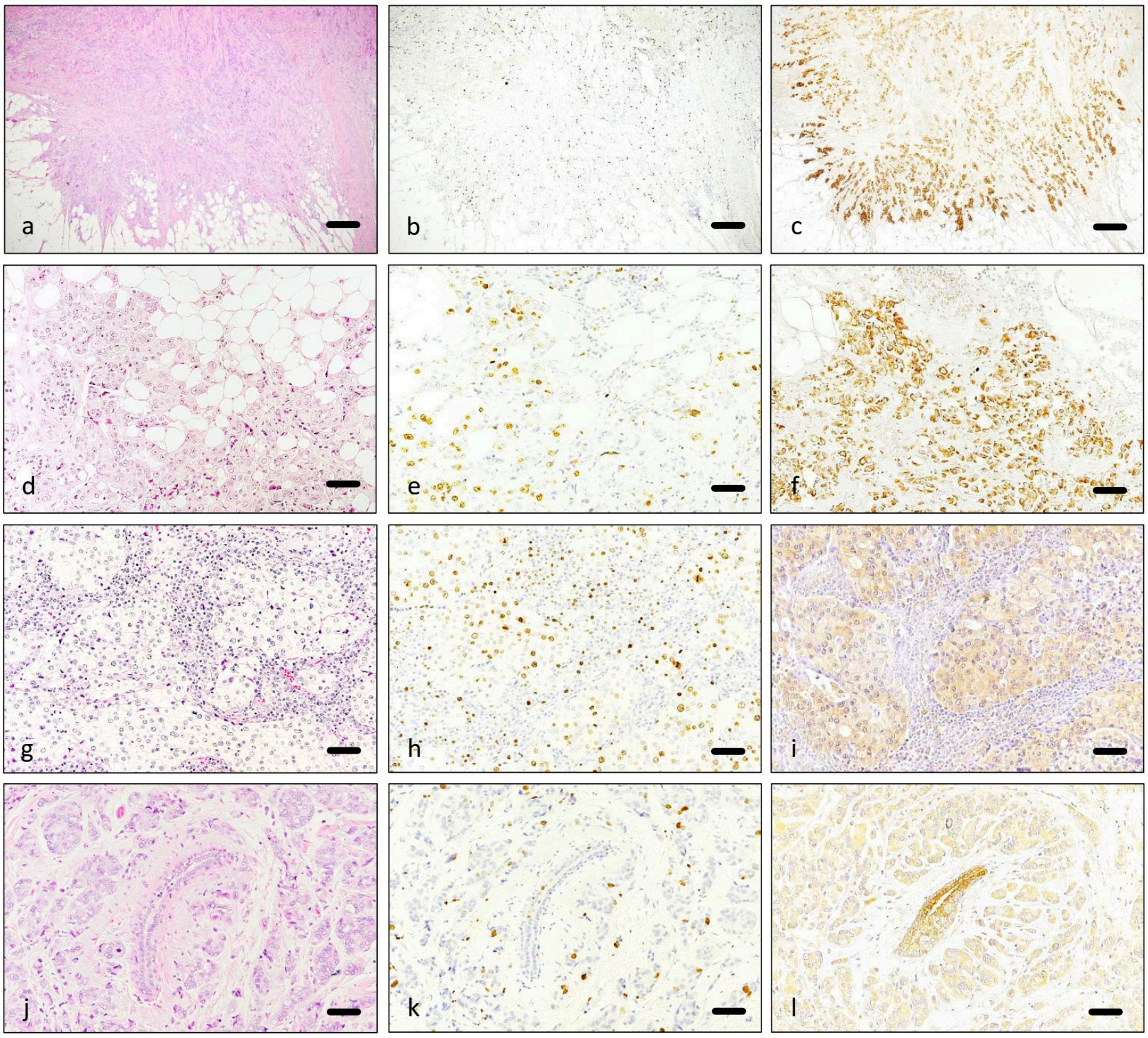

Figure S1. Consecutive sections of breast carcinoma stained with hematoxylin-eosin (**a,d,g,j**) and immunostained with Ki67 (**b,e,h,k**) and Piezo2 (**c,f,i,l**). Although Piezo2 has commonly an homogeneous pattern of immunostaining, sometimes there is certain variation in the intensity of different tumoral regions, illustrated in image c, where the invasive front has a 3+ intensity while the more central part has 2+. Scale bar 250 µm (**a–c**) and 50 µm (**d–l**).
